# Notch1 Signaling Contributes to Mechanical Allodynia Associated with Cyclophosphamide-Induced Cystitis by Promoting Microglia Activation and Neuroinflammation

**DOI:** 10.1155/2021/1791222

**Published:** 2021-10-04

**Authors:** Jialiang Chen, Honglu Ding, Bolong Liu, Xiangfu Zhou, Xin Zhou, Zhijun Lin, Fei Yang, Hailun Zhan, Hengjun Xiao

**Affiliations:** ^1^Department of Urology, The Third Affiliated Hospital of Sun Yat-sen University, Guangzhou 510630, China; ^2^Sun Yat-sen University Cancer Center, State Key Laboratory of Oncology in South China, Collaborative Innovation Center for Cancer Medicine, Guangzhou 510060, China; ^3^Department of Anesthesiology, Guangdong Provincial People's Hospital, Guangdong Academy of Medical Sciences, Guangzhou 510080, China

## Abstract

**Aims:**

Notch1 signaling regulates microglia activation, which promotes neuroinflammation. Neuroinflammation plays an essential role in various kinds of pain sensation, including bladder-related pain in bladder pain syndrome/interstitial cystitis (BPS/IC). However, the impact of Notch1 signaling on mechanical allodynia in cyclophosphamide- (CYP-) induced cystitis is unclear. This study is aimed at determining whether and how Notch1 signaling modulates mechanical allodynia of CYP-induced cystitis.

**Methods:**

CYP was peritoneally injected to establish a bladder pain syndrome/interstitial cystitis (BPS/IC) rat model. A *γ*-secretase inhibitor, DAPT, was intrathecally injected to modulate Notch1 signaling indirectly. Mechanical withdrawal threshold in the lower abdomen was measured with von Frey filaments using the up-down method. The expression of Notch1 signaling, Iba-1, OX-42, TNF-*α*, and IL-1*β* in the L6-S1 spinal dorsal horn (SDH) was measured with Western blotting analysis and immunofluorescence staining.

**Results:**

Notch1 and Notch intracellular domain (NICD) were both upregulated in the SDH of the cystitis group. Moreover, the expression of Notch1 and NICD was negatively correlated with the mechanical withdrawal threshold of the cystitis rats. Furthermore, treatment with DAPT attenuated mechanical allodynia in CYP-induced cystitis and inhibited microglia activation, leading to decreased production of TNF-*α* and IL-1*β*.

**Conclusion:**

Notch1 signaling contributes to mechanical allodynia associated with CYP-induced cystitis by promoting microglia activation and neuroinflammation. Our study showed that inhibition of Notch1 signaling might have therapeutic value for treating pain symptoms in BPS/IC.

## 1. Background

Bladder pain syndrome/interstitial cystitis (BPS/IC) is a syndrome complex characterized by bladder-related pain associated with lower urinary tract symptoms, mainly urinary frequency and urgency [1]. A clinical cohort study revealed that most BPS/IC female patients suffer from constant pain for more than five years [2]. As the dominating symptom of BPS/IC, unrelieved chronic pain can potentiate severe psychological comorbidities, including depression and anxiety, which further aggravate a patient's quality of life [3]. However, the mechanism of bladder-related pain is still unclear, accounting for the limited therapeutic strategies for BPS/IC. Therefore, it is of great clinical importance to clarify the mechanism based on which to provide therapeutic targets for BPS/IC.

Microglia have been widely recognized as a critical cellular mediator in neuroinflammation. The activated microglia can produce more proinflammatory cytokines such as TNF-*α* and IL-1*β*. The cytokines, on the one hand, induce positive feedback to activate more glial cells and, on the other hand, sensitize surrounding neurons. The inflammatory cycle modulated by microglia ultimately leads to central sensitization and pain response [4]. As mentioned in our previous study, neuroinflammation is a crucial mechanism involved in both the initial and maintaining periods of bladder-related pain in a cystitis animal model [5]. Cui and colleagues also found a similar neuroinflammatory phenomenon in a cystitis mouse model [6]. Moreover, lidocaine [7] can be used to treat BPS/IC patients due to not only its analgesic effect but also its antineuroinflammation effect. Accordingly, glial cells and inflammatory cytokines are the underlying therapeutic targets for BPS/IC.

Notch is a membrane receptor that transduces short-range signals from neighboring cells through interacting with a membrane-bound ligand, Delta-like or Jagged in humans. After cleavage by a *γ*-secretase complex, the intracellular Notch receptor domain (NICD) is released and then translocates to the nucleus where it binds to transcription factors and modulates cell proliferation, differentiation, and cell death [8]. Notch1, as one of the four mammalian homologs of Notch, can orchestrate microglia activation contributing to neuroinflammation [9–11]. Furthermore, inhibition of Notch1 could attenuate neuropathic pain through alleviating neuroinflammation [12, 13]. However, whether and how Notch1 signaling modulates mechanical allodynia in CYP-induced cystitis is unknown. This study is aimed at determining the role and mechanism of Notch1 signaling on the initiation and development of mechanical allodynia in a CYP-induced cystitis rat model.

## 2. Materials and Methods

### 2.1. Animals and Husbandry

Female Sprague Dawley rats (200–220 g, Laboratory Animal Center, Sun Yat-sen University, Guangzhou, China) were used and housed in a temperature-controlled room (24 ± 1°C) under a standard 12/12 h light/dark cycle with access to food and water ad libitum. All experimental procedures were approved by the Animal Care Committee of Sun Yat-sen University and conducted under the guidelines of the National Institutes of Health on animal care and ethical guidelines.

### 2.2. Drugs

Cyclophosphamide (CYP, 50 mg/kg; Sigma, St. Louis, MO) was intraperitoneally injected every three days for seven days (injection on days 1, 4, and 7; three times in total) to establish the cystitis rat model. Intrathecal injection of a *γ*-secretase inhibitor, DAPT (MedChemExpress, Princeton, NJ), was used to inhibit the release of NICD and Notch1 signaling transduction.

### 2.3. Experimental Design

We set up two parts of experiments to verify the effect of Notch1 signaling on mechanical allodynia of the CYP-induced cystitis model. In part 1, animals were randomly divided into three groups (*n* = 8 per group): cystitis animals in the CYP+DAPT group were i.t. with 10 *μ*l 50 *μ*M DAPT (dissolved in 4% DMSO) [14] the day after every CYP injection (i.t. on days 2, 5, and 8; three times in total). Cystitis animals in the CYP group and normal animals in the saline group were both treated with 4% DMSO on the same day of DAPT injection. And the saline group also received the same dose of saline (i.p.) as CYP used in the CYP group.

In part 2, three groups of animals were included (*n* = 10 per group). The CYP+DAPT group was treated with DAPT for three consecutive days from the day after the last CYP injection (days 8, 9, and 10; three times in total). CYP and CON groups were treated with 4% DMSO on the same day of DAPT injection.

### 2.4. Intrathecal Injection

Intrathecal injection was performed as described previously [15]. Under anesthesia, a 25 G needle connected to a 25 *μ*l Hamilton syringe was inserted percutaneously into the vertebral canal between L5 and L6. Tail-flick reaction indicates a successful puncture. In our previous study, intrathecal injection of 20 *μ*l of 1% Chicago Sky Blue showed that the drug was able to reach the L6-S1 spinal cord level.

### 2.5. von Frey Test

Measurement of the lower abdominal mechanical allodynia threshold was widely used as a substitute for vesical pain in the CYP-induced cystitis model [16]. And a Dixon up-down method with von Frey filaments (rated at 0.4, 0.6, 1, 2, 4, 6, 8, and 15 g) was used to measure the evoked pain threshold in the lower abdomen. In brief, animals were placed in separate plexiglass chambers positioned on a mesh table and acclimatized to the chamber environment for 30 min before testing. To avoid desensitization, different lower abdominal areas were stimulated. The test started with a dose of 2 g, and the next stronger or weaker filament was applied after a negative or positive response, respectively, was elicited. Positive responses include “licking behavior” and “freezing behavior”.

### 2.6. Western Blotting

Under anesthesia, the L6-S1 spinal dorsal horn (SDH) was quickly harvested from rats. The supernatant was collected and then frozen at -80°C after the tissue samples were mechanically homogenized and centrifuged. Samples were homogenized in a RIPA lysis buffer containing proteinase and phosphatase inhibitors. Proteins in the supernatant were separated by sodium dodecyl sulfate-polyacrylamide gel electrophoresis and then transferred onto polyvinylidene fluoride membranes (Millipore, Billerica, MA, USA). 5% bovine serum albumin solution was used to block the membranes for 60 min at 37°C. After that, the blots were incubated with primary antibody for Notch1 (1 : 500; Santa Cruz Biotechnology, Dallas, TX), NICD (1 : 500; Abcam, Cambridge, UK), ionized calcium-binding adapter molecule 1 (Iba-1, 1 : 1000; Abcam), phosphor-p38 (1 : 1000; Cell Signaling Technology, Danvers, MA), TNF-*α* (1 : 1000; Bioworld Technology, Inc., Louis Park, MN, USA), IL-1*β* (1 : 2500; Abcam), and *β*-actin (1 : 1000; Cell Signaling Technology, Danvers, MA) overnight at 4°C. Secondary antibodies conjugated with horseradish peroxidase (1 : 10,000; KPL, SeraCare, Milford, MA, USA) were applied, and the membrane was incubated for 1 h. Immune complexes were detected with an enhanced chemiluminescence liquid (Millipore). A computer-assisted imaging analysis system (ImageJ; National Institutes of Health, Bethesda, MD, USA) was used to quantify the band intensities.

### 2.7. Immunofluorescence

Under anesthesia, rats were perfused with 4% paraformaldehyde through the ascending aorta. The L6-S1 spinal cord section was removed and postfixed in paraformaldehyde for 30 min. The spinal cord was then transferred to 30% sucrose for dehydration at 4°C. Tissues were sectioned (25 *μ*m thickness) and processed for immunofluorescence staining. Sections were blocked for 1 h and then incubated with primary antibodies against Iba-1 (1 : 200; Abcam), CD11b (OX-42, 1 : 400; Abcam), TNF-*α* (1 : 200; Bioworld), and IL-1*β* (1 : 500; Abcam), overnight at 4°C. After that, the sections were incubated in Cy3 or Alexa-488 conjugated secondary antibodies (Jackson Laboratories, Bar Harbor, ME, USA) for 1 h at room temperature. A Leica fluorescence microscope (Leica DFC350 FX camera; Wetzlar, Germany) was used to measure and image the stained section. To quantify Iba-1, OX-42, TNF- *α*, and IL-1*β* expression in the L6-S1 SDH, the fluorescence intensity of each area was analyzed with ImageJ software. To verify the specificity of primary antibodies, immunostaining was also performed in parallel but without primary antibodies (data not shown).

### 2.8. Statistical Analysis

All data are expressed as the mean ± standard error of the mean (SEM). SPSS 21.0 (SPSS, Inc., Chicago, IL, USA) was used to perform data analyses. The results from Western blotting and immunofluorescence were analyzed with a one-way analysis of variance (ANOVA) followed by the Tukey post hoc test. For the von Frey test, the data were statistically analyzed using a repeated-measure two-way ANOVA followed by a Tukey post hoc test. The Shapiro-Wilk test was used to verify normal distribution of the data before each ANOVA test. Linear regression analysis was performed, and the correlation coefficients were calculated to determine the relationship between the Notch1/NICD expression and the mechanical withdrawal threshold. Differences with *P* < 0.05 were considered statistically significant.

## 3. Results

### 3.1. Notch1 Signaling Was Upregulated in the SDH of the CYP-Induced Cystitis Model

**Figure 1:**
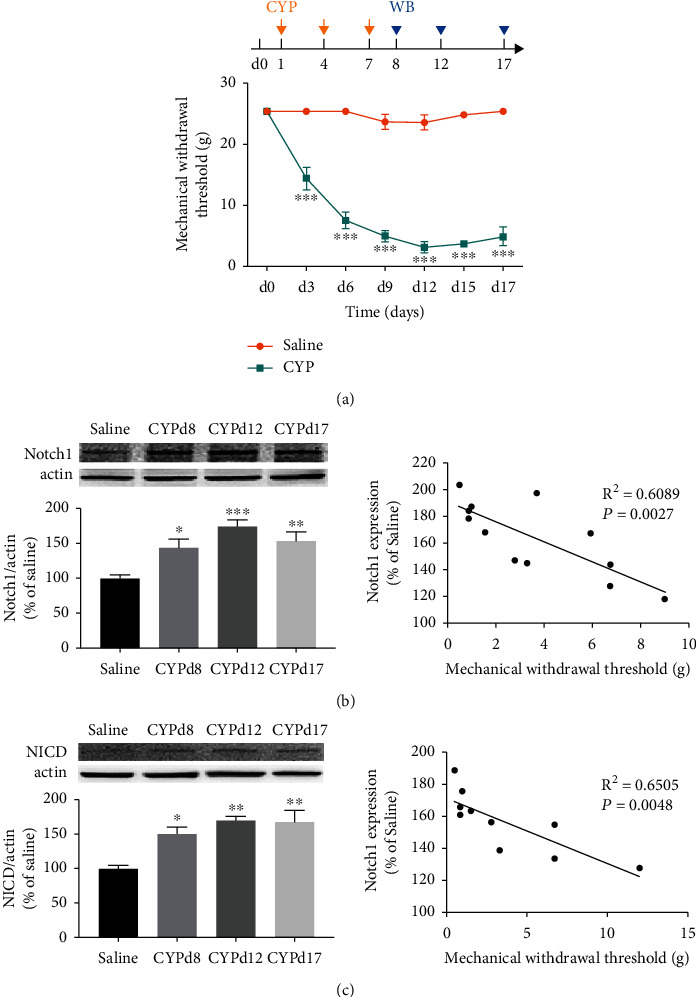
Notch1 signaling was upregulated in the spinal dorsal horn (SDH) of the CYP-induced cystitis model. (a) The mechanical withdrawal threshold of the CYP-induced cystitis model was significantly reduced since the first CYP injection and remained for at least 17 days. *n* = 15 per group. The data were analyzed by two-way ANOVA followed by the Tukey post hoc test. (b) The expressions of Notch1 protein were significantly upregulated at all three time points (days 8, 12, and 17 after the onset of CYP injection). And the expression of Notch1 was negatively correlated with the mechanical withdrawal threshold of the cystitis animals. *n* = 5 per group. (c) The expressions of NICD protein were significantly upregulated at all three time points. And the expression of NICD was also negatively correlated with the mechanical withdrawal threshold of the cystitis animals. *n* = 4 per group. Data in (b) and (c) were analyzed by one-way ANOVA followed by the Tukey post hoc test and linear regression. The correlation coefficients were calculated. ^∗^*P* < 0.05, ^∗∗^*P* < 0.01, and ^∗∗∗^*P* < 0.001 vs. saline group.

As shown in Figure 1(a), the mechanical withdrawal threshold of the CYP-induced cystitis model was significantly reduced after the first CYP injection (*P* < 0.001) and maintained for at least 17 days. The Western blotting analysis results in Figure 1(b) showed that when compared with the saline group, Notch1 in L6-S1 SDH was significantly overexpressed in CYP-induced cystitis group at the three time points, days 8, 12, and 17 after the first CYP injection (*P* < 0.05 for d8, *P* < 0.001 for d12, and *P* < 0.01 for d17). Additionally, NICD was also significantly upregulated in the cystitis group at the time points (Figure 1(c); *P* < 0.05 for d8, *P* < 0.01 for d12 and d17). Furthermore, to confirm the correlation between the Notch1 expression in SDH and the mechanical withdrawal threshold of the cystitis model, we performed a linear regression analysis. We can see in Figures 1(d) and 1(e) that the expression of Notch1 and expression of NICD were both negatively correlated with the mechanical withdrawal threshold in the cystitis group.

### 3.2. Inhibition of Notch1 Signaling Attenuated Mechanical Allodynia of CYP-Induced Cystitis Animals

**Figure 2:**
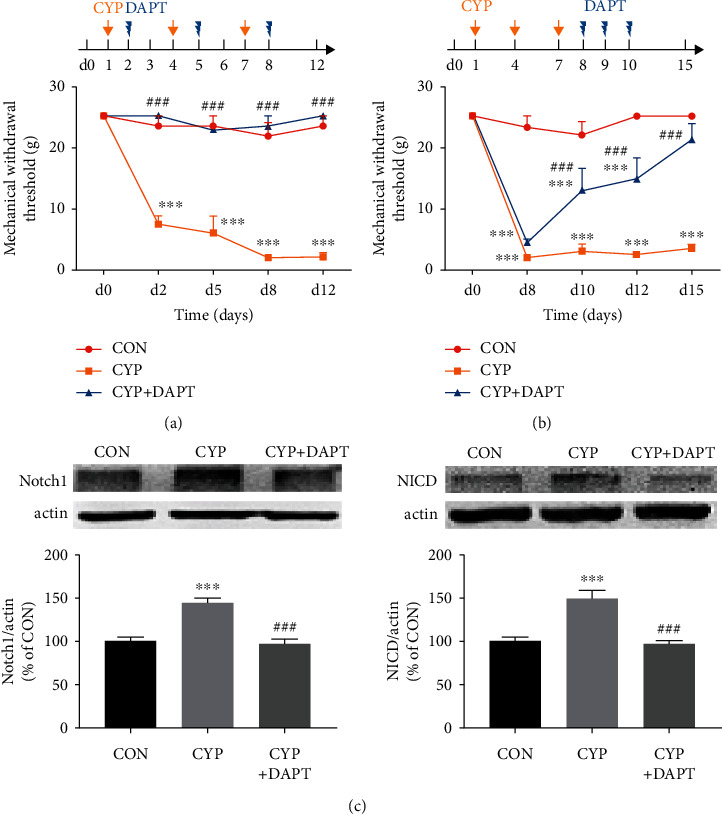
Inhibition of Notch1 signaling attenuated mechanical allodynia in the CYP-induced cystitis model. (a) Intrathecal injection of DAPT (a *γ*-secretase and Notch1 signaling inhibitor) the day after every CYP injection could reverse the reduced mechanical withdrawal threshold of cystitis animals to the basal level. In comparison, the mechanical withdrawal threshold of the CYP-induced cystitis animals without DAPT treatment persisted at a low level for at least 12 days. *n* = 8 per group. (b) Intrathecal injection of DAPT after the establishment of the CYP-induced cystitis model for three consecutive days could gradually reverse the decrease of mechanical withdrawal threshold in the cystitis animals. In comparison, the threshold of the cystitis animals without DAPT treatment persisted at a low level for at least 15 days. *n* = 10 per group. The data in (a) and (b) were analyzed by two-way ANOVA followed by the Tukey post hoc test. (c) The overexpression of Notch1 and NICD in the SDH of cystitis animals in (b) was neutralized by DAPT treatment. *n* = 4 per group. Data were analyzed by one-way ANOVA followed by the Tukey post hoc test. ^∗∗∗^*P* < 0.001 vs. CON group. ^###^*P* < 0.001 vs. CYP group.

After validating the upregulation of Notch1 signaling in SDH of cystitis animals, we identified whether inhibition of Notch1 signaling could attenuate mechanical allodynia of CYP-induced cystitis. We designed a two-part experiment, as mentioned previously in Materials and Methods. The results were shown in Figures 2(a) and 2(b).

The treatment of DAPT the day after every CYP injection for three doses in total could significantly reverse the decreased allodynia threshold of cystitis animals to a CON level. However, the allodynia threshold of the CYP group with a DMSO treatment remained significantly reduced since the first CYP injection. In the case of DAPT treated for three consecutive days after the last CYP injection, the mechanical withdrawal threshold of the CYP+DAPT group was significantly increased compared to the CYP group with DMSO treatment on day 10 (after two times of DAPT injection; *P* < 0.001). However, the mechanical withdrawal threshold of the CYP+DAPT group was still significantly lower than that of the CON group (*P* < 0.001). Furthermore, the mechanical withdrawal threshold of the CYP+DAPT group was gradually increased over time, and on day 15, no significant difference was shown between the CYP+DAPT group and the CON group (*P* > 0.05).

We then operated Western blotting analysis to confirm the inhibition of Notch1 signaling after DAPT treatment. The L6-S1 SDH sample was harvested from the three groups of animals used in Figure 2(b). Results indicated that upregulation of both Notch1 and NICD was significantly inhibited to a CON level by DAPT treatment (*P* < 0.001).

### 3.3. Activation of Microglia in SDH Was Suppressed by Inhibition of Notch1 Signaling with DAPT Treatment

After identifying whether Notch1 signaling can modulate mechanical allodynia of cystitis animals, we explored the underlying mechanism of Notch1 signaling promoting mechanical allodynia. As mentioned in the background, microglia activation could be involved in the process.

**Figure 3:**
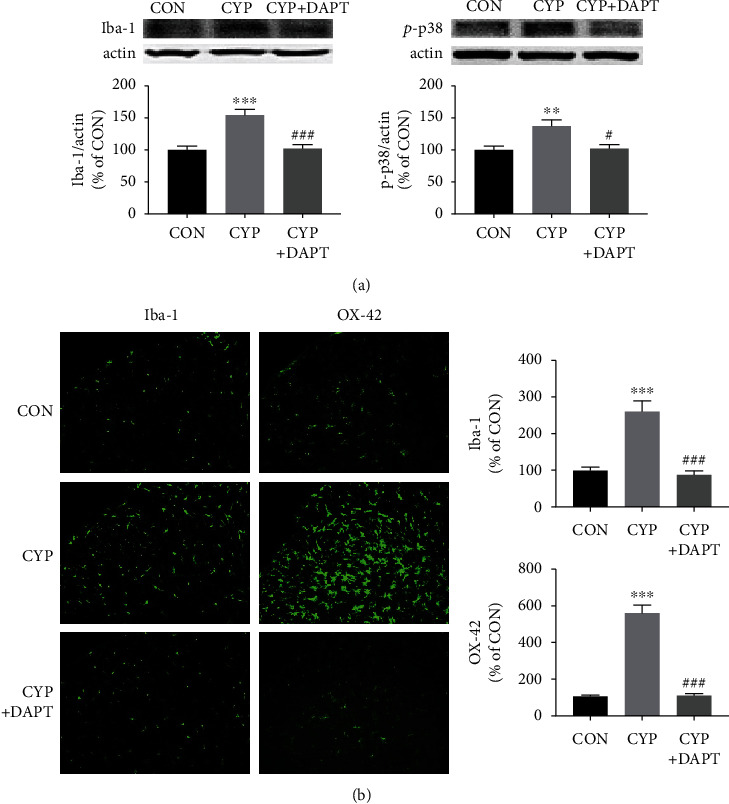
Inhibition of Notch1 signaling suppressed microglia activation in the SDH of the CYP-induced cystitis model. (a) Western blotting analysis showed that intrathecal injection of DAPT significantly neutralized the overexpression of Iba-1 and *p*-p38, which are markers of microglia activation. (b) Immunofluorescence staining also showed that intrathecal injection of DAPT significantly neutralized the overexpression of microglia activation markers, Iba-1 and OX-42. *n* = 5 per group. ^∗∗^*P* < 0.01 and ^∗∗∗^*P* < 0.001 vs. CON group; ^#^*P* < 0.05 and ^###^*P* < 0.001 vs. CYP group. Data were analyzed by one-way ANOVA followed by the Tukey post hoc test.

Expression of the markers of microglia activation, Iba-1 and OX-42, was measured by Western blotting analysis and immunofluorescence. The results of Western blotting analysis and immunofluorescence indicated that the overexpression of Iba-1 and OX-42 in SDH of cystitis animals was reversed by DAPT application (Figures 3(a) and 3(c)). We also detected another indirect marker for microglia activation, *p*-p38 [17], to further validate the influence of DAPT on microglia activation. As shown in Figure 3(b), DAPT treatment could significantly reverse the upregulation of *p*-p38 in SDH of cystitis animals (*P* < 0.05). The expression changes of the three microglia activation markers after DAPT treatment were consistent.

### 3.4. Inhibition of Notch1 Signaling with DAPT Suppressed TNF-*α* and IL-1*β* Production in the SDH of the Cystitis Model

**Figure 4:**
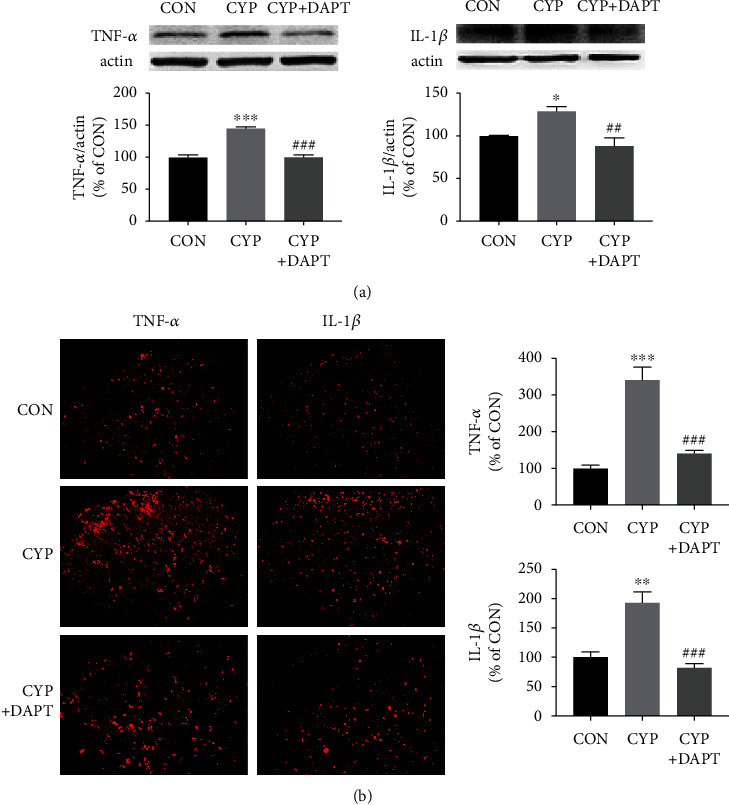
Inhibition of Notch1 signaling suppressed TNF-*α* and IL-1*β* production in the SDH of CYP-induced cystitis model. (a) Western blotting analysis showed that intrathecal injection of DAPT significantly inhibited the production of the two critical proinflammatory cytokines, TNF-*α* and IL-1*β*. (b) Immunofluorescence staining also showed that the production of TNF-*α* and IL-1*β* was inhibited by intrathecal injection of DAPT. *n* = 5 per group. ^∗^*P* < 0.05, ^∗∗^*P* < 0.01, and ^∗∗∗^*P* < 0.001 vs. CON group; ^##^*P* < 0.01 and ^###^*P* < 0.001 vs. CYP group. Data were analyzed by one-way ANOVA followed by the Tukey post hoc test.

TNF-*α* and IL-1*β* are two crucial proinflammatory cytokines that can be produced and released by activated microglia [18]. Since microglia activation was validated to be inhibited by DAPT treatment, we then evaluated whether the production of TNF-*α* and IL-1*β* can also decrease after DAPT treatment. Results of Western blotting analysis and immunofluorescence in Figure 4 indicated that TNF-*α* and IL-1*β* were both overexpressed in the SDH of the CYP group with DMSO treatment. And DAPT could significantly reverse the overexpression of the two proinflammatory cytokines to a CON level.

## 4. Discussion

In our present study, we revealed that Notch1 signaling was upregulated in the L6-S1 SDH of the CYP-induced cystitis rat model, and the expression of Notch1 and NICD was negatively correlated with the mechanical withdrawal threshold of the cystitis animals. Furthermore, treatment with DAPT during or after the cystitis model establishment could inhibit the overexpression of Notch1 signaling. And inhibition of Notch1 signaling by DAPT contributed to the attenuation of mechanical allodynia of the cystitis model. Moreover, DAPT could inhibit microglia activation and reverse the upregulation of TNF-*α* and IL-1*β* in cystitis animals. Taken together, our study indicated that Notch1 signaling promoted microglia activation, contributing to mechanical allodynia of CYP-induced cystitis.

As mentioned in the background, the Notch signaling pathway plays a vital role in cell proliferation, differentiation, and cell death. Recently, Notch1 signaling has been revealed to modulate neuropathic pain via regulating neuroinflammation [12, 19]. Although visceral pain is different from neuropathic pain to some extent, we found in our previous studies [5, 20, 21] that neuroinflammation in the central nervous system including SDH and the hippocampus could also be an underlying mechanism of bladder-related pain in a cystitis rat model. To be specific, the production of proinflammatory factors evoked by microglia activation could be an essential mechanism of both bladder-related pain and neuropathic pain.

Interestingly, Notch1 signaling was identified as a contributor to microglia activation [9, 22]. Therefore, we speculated that Notch1 signaling could regulate microglia activation in SDH, contributing to the mechanical allodynia of the CYP-induced cystitis model. And our present study is the first work to testify to this speculation. Moreover, the intracellular domain of Notch1 receptor, NICD, was also found to be upregulated in the cystitis model in our study. The overexpression of the total Notch1 receptor could account for the upregulation of NICD. However, we could not exclude the possibility that more *γ*-secretase was also activated to release more NICD from the Notch1 receptor.

We were also curious about how Notch1 signaling activated microglia in the SDH of the cystitis model. However, we could not directly testify whether Notch1 or NICD was expressed by microglia. Unfortunately, no study right now can demonstrate the colocalization of the Notch1 receptor in SDH tissue. Although an in vitro study showed that microglial cells express the Notch1 receptor [9], it could be totally different from the circumstances of in vivo studies. In our present study, inhibition of Notch1 signaling could suppress the activation of microglia. The microglia activation could be inhibited in two different ways. One is that the Notch1 receptor is located at the microglia membrane, and inhibition of Notch1 can directly downregulate target genes related to microglia activation. Another is that inhibition of Notch1 suppressed the activation of other glial cells or neurons, leading to the deactivation of microglia. A previous study revealed that reactive astrocytes could express NICD1 and could also be regulated by Notch1 after stroke [23]. Another work, on the other hand, showed Notch1 signaling promoted TNF-*α* production in neurons of diabetic neuropathic rats, as an essential mechanism in diabetic neuropathy [12]. More research is needed to clarify the mechanism of Notch1 signaling modulating microglia activation. Besides, whether and how Notch1 modulates other glial cells and neurons in a cystitis model also need to be addressed.

Additionally, in our present study, the changes of the mechanical withdrawal threshold induced by two different applications of DAPT were different. DAPT applied during the establishment of the cystitis model could prevent the onset of mechanical allodynia in cystitis animals. However, if DAPT was applied after the establishment of the cystitis model, the mechanical withdrawal threshold of the cystitis animals needs about one week (from d8 to d15) to return to the basal level. The outcome of DAPT treatment could depend on the expression level of the proinflammatory cytokines. It is widely accepted that glial cells can interact with neurons through the release of mediators including chemokines, cytokines, and growth factors. The interaction between glial cells and neurons is a critical mechanism contributing to central sensitization and pain sensitization [24]. The establishment of the cystitis model in our present study needs three doses of CYP injections lasting one week, during which proinflammatory cytokines including TNF-*α* and IL-1*β* were gradually produced and accumulated. If DAPT is applied during the establishment of the cystitis model, it could significantly reverse the overexpression of proinflammatory cytokines to the basal level. However, if no treatment is applied during the establishment of the cystitis model, the overexpression of proinflammatory cytokines could be difficult to reverse because the production of these cytokines has reached a peak level. Furthermore, it also inspires us that the treatment outcome could somehow depend on the disease duration in BPS/IC patients. For example, the treatment outcomes of botulinum toxin A are different between IC/BPS patients with different disease duration [25].

The main limitation of our present study is that we only focus on the Notch1 receptor in Notch1 signaling without involving the ligands of Notch1 signaling, including Jagged and Delta. More research is needed to explore the specific ligands binding to Notch1 signaling and inducing downstream activities, which contribute to mechanical allodynia in our cystitis model. Moreover, we only used one concentration level of DAPT to inhibit Notch1 signaling, and we need to further investigate the effect of different concentration levels of DAPT on mechanical allodynia of cystitis animals.

## 5. Conclusion

In our study, we revealed that the Notch1 receptor and NICD were both upregulated in the L6-S1 SDH of CYP-induced cystitis rats. Furthermore, the expression of the two proteins was negatively correlative with the pain threshold of the cystitis animals. Inhibition of Notch1 signaling with a *γ*-secretase inhibitor, DAPT, could attenuate mechanical allodynia of cystitis animals by suppressing microglia activation as well as overexpression of TNF-*α* and IL-1*β*. Taken together, Notch1 signaling may promote microglia activation and production of TNF-*α* and IL-1*β*, contributing to mechanical allodynia associated with CYP-induced cystitis.

## Data Availability

Data will be available on request.
